# The Nexus Between Plant and Plant Microbiome: Revelation of the Networking Strategies

**DOI:** 10.3389/fmicb.2020.548037

**Published:** 2020-09-04

**Authors:** Olubukola Oluranti Babalola, Ayomide E. Fadiji, Ben J. Enagbonma, Elizabeth T. Alori, Modupe S. Ayilara, Ayansina S. Ayangbenro

**Affiliations:** ^1^Food Security and Safety Niche, Faculty of Natural and Agricultural Sciences, North-West University, Mmabatho, South Africa; ^2^Department of Crop and Soil Sciences, Landmark University, Omu-Aran, Nigeria

**Keywords:** microbiome, network analysis, co-occurrence, plant–microbe-interactions, keystone taxa

## Abstract

The diversity of plant-associated microbes is enormous and complex. These microbiomes are structured and form complex interconnected microbial networks that are important in plant health and ecosystem functioning. Understanding the composition of the microbiome and their core function is important in unraveling their networking strategies and their potential influence on plant performance. The network is altered by the host plant species, which in turn influence the microbial interaction dynamics and co-evolution. We discuss the plant microbiome and the complex interplay among microbes and between their host plants. We provide an overview of how plant performance is influenced by the microbiome diversity and function.

## Introduction

Plants have evolved a plethora of diverse and complex microbial communities, which affect plant growth and health in a beneficial, harmful, or neutral way. These microbial communities display different interactomes, genetic landscape, and information-processing networks (Kozyrovska, 2013). Plant controls its interactions with microbes and the result of this interaction depends on the interacting partners and their environments. Although, there is considerable amount of research outputs focusing on the dynamics, structure and functional roles of plant microbial communities, the mechanisms of interactions and processes driving the modulation of the plant microbiome are still largely unclear. This makes it difficult to understand the key ecological processes that control the entire microbial community structure (Toju et al., 2016).

The microbiota of plant is diverse and complex. The metabolism of individual members of the microbiota are often linked together in a way that the community aggregate can be considered to possess a ‘net’ metabolism. This net metabolism is the volatile signature that propagates their ecological network and allows a non-invasive analysis of active microbiota (Redeker et al., 2018). Ecological network analysis that describes species interactions and strength of their interactions provides unprecedented opportunities in understanding the underlying principles of plant–microbe interactions within a community, impacts of environmental change, the ability to quantify ecosystem services, and identification of keystone taxa (Derocles et al., 2018; Bennett et al., 2019). Deciphering the interactions between plant-associated microbes are important in understanding their structure and function, and how trait-associated microbiomes influences the host plant.

Network analyses provide co-variation and non-random patterns, which show the organization of a community, such as direct interactions or shared niches or guilds, and provide tools for examining ecological concepts (Shi et al., 2016). Thus, giving insight on how information flows among members of the microbiome or its environment (Jiang et al., 2019). Network analyses use different techniques to determine the taxa that compete with one another, those that co-depend on one another or the keystone populations in a community. Its application in plant microbiome studies can be used to model the co-occurrence of microbes, unearth relationships important for community assembly or stability and insight on the influence of different interactions on plant health (Layeghifard et al., 2017).

Co-culturing experiments have been used to study interactions between two microbes and such experiments have been used in observing the effects of each other’s growth and physiology (Garcia and Kao-Kniffin, 2019). While co-culturing has been the preference, rapid advancement in high-throughput sequencing has provided a revolutionary tool for studying multiple and complex interactions between microorganisms and their host plant. High-throughput sequencing is increasingly being used to infer linkages between microbial groups that jointly build up a community. This will help to decipher and predict the functional roles, shared physiologies and habitat affinities (Jiang et al., 2017).

Despite the advances in high-throughput sequencing, their application remains nascent, and the inferred interactions should be interpreted with caution (Layeghifard et al., 2017). Unraveling the relationships between diverse microbial species and their functions will facilitate the understanding of their interactions within the plant microbiota. In this review, we highlight the plant microbiome and the interplay among plant microbiota and host plants. We equally provide an overview of how plant performance is influenced by the microbiome diversity and function.

## The Plant Microbiome

Plants are shaped by diverse types of microorganisms playing notable functions in plant growth and health enhancement. Reports from the last decade have affirmed that plants and plant organs possess nexus microbial assemblages associated with it (Hardoim et al., 2015; Reinhold-Hurek et al., 2015). The microbial constituent of plant holobiont is called plant microbiota (consisting all microbes) or the plant microbiome (accounting for all the microbial genomes). They inhabit the endosphere, phyllosphere, and the rhizosphere with beneficial roles in plant growth promotion and health (Brader et al., 2017; Lemanceau et al., 2017). Unveiling the functions performed by these plant-associated microbes and the factors affecting the community assembly can provide more insights into plant as a meta-organism and the benefits conferred on the plant by microbial partners (Hardoim et al., 2015; Hacquard, 2016).

The plant microbiome is built by the genotype of plant, plant species, edaphic and other environmental factors, but the roles of this makeup are difficult to separate from each other in natural environments (Compant et al., 2019). We explore the endophytes, epiphytes, and rhizobiome as major examples of organisms inhabiting endosphere, phyllosphere, and rhizosphere of a plant, respectively.

## Endophytes

Endophytes are microbes that successfully colonize the tissue of vascular plants and have been reported to be isolated in virtually all plants (Fadiji and Babalola, 2020b). They are initially known not to be harmless to the host plants and their association with plants can be obligate or facultative (Nair and Padmavathy, 2014). A recent research by Brader et al. (2017) revealed that endophytes can also be defined in terms of their ecological niche and not only the function they perform in the host. On this basis, endophytes were found to be either pathogenic or non-pathogenic. Most endophytic microbes do not show any harmful effects on a few plant species; however, when tested on other plants, they may be pathogenic. The pathogenicity attribute of endophytes can be linked to several of biotic interactions and environmental factors. For example, fluorescent Pseudomonads, have been reported to be pathogenic to the leatherleaf plant under certain conditions even though studies have found the organism to be beneficial to most plant species (Kloepper et al., 2013). Nevertheless, endophytes have been observed to be active in biocontrol of phytopathogens, plant growth enhancement, and in the secretion of metabolites of great biotechnological or pharmaceutical importance (Sharma et al., 2017).

The endophytic association can be carried by archaea, bacteria, and fungi but endophytic bacteria and fungi are the most prominent (Patle et al., 2018). They live symbiotically with most plants by entering their cells (Fadiji and Babalola, 2020b). There exists a wide diversity of endophytes, mostly with a great improvement in their ecological roles alongside the production of numerous secondary metabolites. Endophytes were reported to be naturally resident in many host plants (Suryanarayanan, 2013). Different endophytes can be found in different parts of a plant mainly in the stem, leaves or roots (Fürnkranz et al., 2012). Most endophytes that are found in vascular plants were discovered to maintain a *symbiotic* interaction. The endophytes obtained their nutrients from host plants and consequently contribute significant benefits to the growth and health of host plants. These endophytes harmlessly live within the tissues of the host they have colonized, thereby facilitating an indirect defense against herbivores (Bamisile et al., 2018).

Endophytes receive nutrition as well as protection from the host while encouraging the absorption of nutrients and protection of the host from abiotic and biotic stresses and pests (Omomowo and Babalola, 2019). It has also been reported that the availability of endophytes affects the health, growth and development of plant, and different types of plant communities, ecosystem functioning, and population dynamic (Hardoim et al., 2015). Many endophytic microbes have been reported to have developed gradually, finding their ways into the plant, and as this association continues, they devise new ways to inhabit, evolve, establish, and improve the association they have established with the host (Goyal et al., 2016). However, high-throughput sequencing insights into the structure and function of endophytic microbes can help in understanding the community network, discovering novel genes and roles performed by these organisms in enhancing plant growth and health.

## Epiphytes

Epiphytes are microbes that inhabit and multiply upon a living plant for support. They are not parasites, but rely on the plant for nutrition and water. Epiphytism is exhibited by many microbial groups some of which are algae, bacteria, protozoa, nematodes, fungi, and plants (Lindow and Brandl, 2003). Epiphytes are also part of the makeup of plant microbiome, and consist of organisms that colonize the external surface of plant tissue (phyllosphere). Although epiphyte can be used to describe the external area of plants, it is commonly used in association with the leaf surface (Vorholt, 2012). Most microbial communities inhabiting the phyllosphere have been implicated in the enhancement of plant growth through nitrogen fixing, plant protection against pathogens, and biosynthesis of plant hormones (Andreote et al., 2014; Berg et al., 2014b). Epiphytes are also notable pieces of global processes, an example is the sequestration of carbon (Bulgarelli et al., 2012), and they have a great prospect in boosting sustainable agriculture. Epiphytes can withstand extreme environmental conditions, known as oligotrophic environment, characterized by limited nutrients, inconsistent humidity, pH, UV radiation, and temperature (Andreote et al., 2014).

The origin of microbes that make up epiphytes is fully known. Bulgarelli et al. (2012) reported that plants are subjected to a high rate of microbial inoculation, enhanced by the activities of wind and vectors. The study further stressed that air and its aerosols, water and soil are the major sources of epiphytes found in the phyllosphere. It is also possible that the community of epiphytic microorganisms is regulated by specific environmental factors (Berg et al., 2014b). Differences in these environmental factors might enhance the diversity, structure, and the abundance of the epiphytic organisms in individual plant species. Redford et al. (2010) reported that different species of plant harbors distinct bacterial communities, which can be attributed to a specific niche and the local environment, influenced by the genotype and functional metabolism of the plant. Geographical distance was also reported to be a major player in the community structure of epiphytic bacteria in grapevines (Bokulich et al., 2014).

## The Rhizobiomes

Rhizobiome is a term used to describe all the microbial communities inhabiting the rhizosphere (Sasse et al., 2018; Olanrewaju et al., 2019). Research has long revealed that plant root exudates attract beneficial microbes to its rhizosphere, however, uninvited ones are also attracted (Olanrewaju et al., 2019). Communities of microbes present in the soil are affected by many factors, which include soil texture and environmental factors (Bach et al., 2018). This study also suggests that the root exudates performs a major function in the abundance and diversity of rhizobiome. Although, Dennis et al. (2010) reported that root exudates perform a considerably limited function in influencing the microbial communities in the rhizosphere compared to the remaining rhizodeposits (mucilages, lysates, sloughed-off root cells, and volatiles). The argument was also further strengthened by a similar study performed on ryegrass (Lettice, 2019).

However, Sasse et al. (2018) reviewed many literature and concluded that plant rhizobiomes are most times (but not in all cases) indifferent from similar plant species and from bulk soil. The authors described plants in this category as those having weak rhizospheric effect (Sasse et al., 2018). Also, Chen et al. (2016) conducted a study to assess the rhizobiome of ryegrass and observed that the abundance of some notable bacterial genera such as *Pseudomonas, Methylobacterium, Rhizobium, Enterobacter*, and *Stenotrophomonas* were more in the endosphere, rhizosphere, and rhizoplane compared to the external rhizosphere. Knowing fully well that various parts of the plant root system secretes diverse types of metabolites (Tückmantel et al., 2017) and that the part called root tips produced the most abundant root exudates (Pausch and Kuzyakov, 2011). It is therefore no longer new that the mature roots and root tips have diverse community of microbes attached to them (Massalha et al., 2017). Saleem et al. (2018) examined the impact of root architecture on plant microbiome and rhizosphere and concluded that root phenotypes, such as density, root length, volume, biomass, and surface area create different ecological niches for some microorganisms to enhance beneficial interactions in the rhizosphere. The study emphasized that since the first part of the plant to make contact with the bulk soil is the root tips, the rhizodeposits secreted and the rhizobiomes linked with them are significant in sustaining the rhizosphere.

Rhizobiomes have been implicated in the enhancement of plant growth, but the mechanisms have not been fully established due to unavailability of required techniques, tools, and low interest in the scientific world (Olanrewaju et al., 2019). However, the introduction of next-generation sequencing techniques such as metagenomics, proteomics, transcriptomics, and metatranscriptomics has helped in exploring the rhizobiomes (Turner et al., 2013; Schlaeppi and Bulgarelli, 2015). However, studies involving the structure, diversity, and function of rhizobiomes are still novel and can be explored to establish their mechanisms of action and contribution toward plant growth and health.

## Factors Affecting Plant Microbiome

Plant microbiome is affected by many biotic and abiotic factors. These factors include salinity, soil moisture, soil organic matter, root exudates, soil type, soil structure, and soil pH (Fierer, 2017). However, factors such as external environmental conditions among which are human practices, presence of pathogens, and climate affect epiphytes and endophytes (Hardoim et al., 2015). Host species attract microorganisms from the rhizosphere, where root exudates, morphology, alongside rhizodeposits perform a major role in the recruitment of plant microbiomes (Hartmann et al., 2009; Chaparro et al., 2014; Reinhold-Hurek et al., 2015).

Studies have revealed that the makeup of the root exudates control the kind of plant-associated microbial community that the plant will attract. Some studies have shown that the exudates secreted by the root of a plant have a major influence on shaping the abundance of rhizospheric microbial communities associated with arboreal and herbaceous plants (Zhang et al., 2014). Shifts in the profile of root exudates have been considered as one of the major drivers of changes in the microbial communities inhabiting the root of plants. An increment in the abundance of rhizospheric bacterial in barley under N growth conditions (Liljeroth et al., 1990), changes in the structure of endophytic bacterial community in sorghum cultivated in nitrogen fertilized and non-fertilized environments (Mareque et al., 2018), alongside the increment in the abundance of some bacterial families in wheat root microbiome (Pagé et al., 2019) were considered. These studies buttressed the fact that changes in the quantity and quality of root exudates under different exposure to nitrogen environment affects the structure of plant microbiome, although the exudates were not fully characterized in those cases. However, the characterization of roots exudates from maize cultivated using increased nitrogen levels was reported by Zhu et al. (2016), the results showed that the total secreted root exudates, such as phenolic compounds, sugar alcohols, and sugars significantly aligned with the level of the fertilizer, which also affected the abundance of root microbiome.

Further studies have also investigated the community function between root-associated microbiomes and root exudates. For instance, a study by Kavamura et al. (2018) revealed that rhizospheric bacteria associated with wheat plants treated without inorganic nitrogen fertilizer enriched the putative functional pathways linked with terpenoid metabolism and reduced number of genes related to the metabolism of carbohydrates and amino acids. This subsequently increased the affected the composition and structure of rhizospheric bacterial communities associated with wheat, especially the phylum Bacteroidetes. Terpenoids are notable examples of root exudates, referred to as nitrification inhibitors (Coskun et al., 2017; Hartman and Tringe, 2019), which control nitrogen loss by nitrification in an environment characterized with low nitrogen. Although further studies are needed in understanding whether the terpenoids secreted by plants are for adaptation, that is nitrifying growth environment or they have some yet to be discovered functions using the inhibition of nitrification as a side effect (Coskun et al., 2017).

Numerous rhizodeposits have also been revealed to influence the composition of plant microbiome (Pascale et al., 2020). The biosynthesis of indolic and aliphatic glucosinolates is part of the defense composition adopted by plants (Xu et al., 2017). Some studies have revealed that aliphatic glucosinolates from root exudates can affect the microbiome in the rhizosphere of a plant (Bressan et al., 2009), while the indolic glucosinolates aggregate in the root of *Arabidopsis* upon attack by pathogens (Bednarek et al., 2005). Furthermore, the combination of exudates secreted by *Arabidopsis* cultivated *in vitro* and applied on soil without the plant showed varied effects of phenolic compounds on the abundance of bacterial groups (Badri et al., 2013).

Another plant/host species growing in the same environment can attract and aggregate different microbiomes to its self from the root compartments and the rhizosphere (Aleklett et al., 2015; Samad et al., 2017). Employing shotgun metagenomic approach and 16S rRNA gene sequencing, Bulgarelli et al. (2015) assessed the root microbiota of different barley species and discovered that the root metabolites and the host innate immune system control the abundance and diversity of the root microbiome. Furthermore, other host-associated factors such as plant developmental stage, plant health, fitness, and age are other notable factors reported to be active in influencing the community structure of host/plant microbiome, especially the bacterial community (Aleklett et al., 2015; Reinhold-Hurek et al., 2015).

Exudates collected from different *Arabidopsis* plants at different plant ages showed variation in sugar levels, which affected microbial functions associated with secondary metabolism and sugar production (Chaparro et al., 2013). In another study, Chaparro et al. (2014) reported that *Arabidopsis* plants at different stages of their development (early and late stages) can influence microbial functions as well as the abundance of Bacteroidetes, Actinobacteria, and Cyanobacteria. Functions similar to pathogens were expressed in the early stage of development while functions associated with chemotaxis and antibiosis were greatly expressed at the late stage of development, indicating a selective pressure during the developmental stages of the plant toward microbes that perform important functions in their host. Similarly, a recent report emphasized that exudates also vary during the growth stages of *Avena barbata*, where sucrose was observed to be high at the early stage of development while defense molecules and amino acids are greatly produced at the late stage of development (Zhalnina et al., 2018).

Furthermore, recent studies have shown that plant genotypes can also influence the abundance of the microbiome associated with the rhizosphere of plants (Haney et al., 2015). The report further revealed that different accessions of *Arabidopsis thaliana* slightly inhibited the species in the family Pseudomonadacea, such as *Pseudomonas syringae*, *P. brassicacearum*, and *P. fluorescens* without having any significant influence on other microbiomes. Therefore, the genotype of a plant is one of the key factors in understanding the abundance of plant-associated bacteria and their role in plant health and physiology with exposure to different abiotic and abiotic environments (Soussi et al., 2016). Similarly, Müller et al. (2015) reported that the genotype of Olive plants has a great influence on the endophyte communities in the leaves of *Olea europaea* L compared to the influence from environmental factors, geographic location and soil types.

Similarly, both land-use history and soil types have been reported to have a higher influence on bacterial communities than plant species (Soussi et al., 2016). In a study by Salles et al. (2004), different plants, such as grass, barley, maize, and oat, were grown under greenhouse with soils having different land use application histories. They reported that land-use history affected the structure of *Burkholderia* community and the diversity of *Pseudomonas*, while showing a great influence on the overall composition of bacterial communities (Salles et al., 2004). In addition, Latour et al. (1996) investigated the diversity of bacteria associated with the root of two plant species. The results showed that both host plant and the type of soil used affected the diversity of bacteria, although soil type was reported as having the most dominant influence (Latour et al., 1996). Furthermore, seasonal variations have been reported to influence the diversity of microbial communities associated with most plants (Soussi et al., 2016). Saul-Tcherkas and Steinberger (2011) investigated the microbial diversity in the rhizosphere of Reaumuria negevensis planted in Negev Desert. The results showed that Actinobacteria was the most abundant phylum in all major seasons except for winter. Although, Acidobacteria had the highest density in the winter while Actinobacteria decreased. Furthermore, phylum Chloroflexi and Bacteroidetes were abundant in summer with a significant reduction in autumn and winter while the abundance of phylum Gemmatimonadetes was reported in autumn (Saul-Tcherkas and Steinberger, 2011).

## Microbiome Network and Interplay

Several literature have shown that plants are inhabited by composite microbial groups and harbor a microbiome. Incipient research work with plants showed that these microbiomes are well organized and form intricate interrelated microbial networks (King et al., 2012; Huang et al., 2020). Inside these networks, each taxon has it specific functions essential for plant health and ecosystem functioning (Zhou et al., 2010). For example, Shi et al. (2016) applied random matrix theory (RMT)-based network analysis of 16S rRNA genes to detect microbial networks linked with *Avena fatua* (wild oat) rhizosphere and reported that increased complexity and connectivity of rhizosphere network are characteristics of the rhizosphere bacterial assemblages. This forms the basic difference between the *Avena fatua* rhizosphere and its bulk soil. This implies that the rhizosphere has more potential for niche-sharing and interactions because rhizosphere networks were significantly more intricate than those in bulk soils.

**FIGURE 1:**
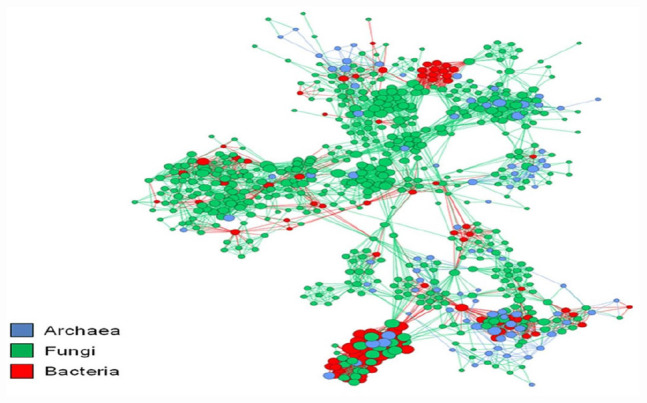
Co-occurrence networks aid in recognizing potential associations between species. The nodes correspond to the microbial operational taxonomic unit and edges to the microbial associations. The figure was adapted from Monard et al. (2016).

The plant microbiome’s functional capability is not the same as the totality of its separate components, as microbial species intensely and regularly interrelate with one another and form a complex network (Khan et al., 2019). Examining huge environmental data produced by high-throughput DNA sequencing tools requires novel investigative methods. To move beyond the rudimentary catalog interpretations of the composition, richness, and variety of microbial assemblages from their natural habitats (Qi et al., 2019). To examine possible relations among microbiome, the major taxon co-occurrence patterns need to be investigated with network exploration (Figure 1). This network analysis can aid in interpreting the organization of intricate microbial groups through space or time (Layeghifard et al., 2017). Network analysis also assists in having a comprehensive insight into the structure and composition of microbial assemblages (Ma et al., 2016). Through an ecological measure based on the checkerboard units (C-score), Barberán et al. (2012) assessed non-random co-occurrence patterns, over 160,000 archaeal and bacterial 16S rRNA gene sequences were collected from 151 soil samples. Their findings revealed a significant non-random co-occurrence pattern with 46.56 C-score when the whole dataset was used. However, the C-score significantly increased to 185.03 when the analysis was restricted to only the operation taxonomic units (OTUs). This form of co-occurrence for microorganisms connotes a non-random community assembly can be a universal characteristic across all forms of life. Furthermore, it indicates the domination of deterministic processes and non-overlapping niches, competitive relationships, or historical effects in determining community structure (Horner-Devine et al., 2007).

To investigate such networks, many tools like Bayesian network approaches (Friedman et al., 2000), differential equation-based network approaches (Akutsu et al., 1999), and relevance/co-expression network approaches (Butte et al., 2000) have been used in genomic ecological studies. Bayesian network approach is a graph-based model of combined multivariate probability distributions that considers characteristics of conditional independence among variables. This approach was first developed to infer gene regulatory networks from steady-state expression data (Friedman et al., 2000) and later expanded to resolve inference problems in time-series expression data. Bayesian network models are smart for their capacity to interpret complex stochastic processes (like networks among genes based on multiple expression measurements) and because they offer a clear method for learning from ‘noisy’ observations (Heckerman et al., 1999). The application of Bayesian network approach was seen in the investigation by Bruex et al. (2012) when they studied the transcriptomes of seventeen root epidermal mutants and two plant hormone treatments. Through the Bayesian network approach, they surmised regulatory interactions among 208 core genes and employed expression information from developmental time-series datasets to place genes sequentially within the network.

Differential equation-based network approach is used to plainly characterize the dependence of the concentration of one gene’s transcripts on that of other genes. Differential equation-based network approaches fall into the model-based approaches that have several algorithms such as singular value decomposition and regression analysis (Yeung et al., 2002). Conversely, the feat of these differential equation-based network approaches has been inadequate because of technical challenges that include the difficulty in assessing the parameters in the differential equation models.

Relevance/co-expression network approaches are useful in finding correlations through disparate biological measures like the RNA expression (Butte et al., 2000). This model has significance for fold differences and it attempts to maximize the number of expressed sequence tags above their threshold. Butte et al. (2001) established that even though RNA expression levels seem to be reliable in duplicate measurements, when whole experiments are duplicated, measured fold differences are not as consistent. Therefore, it is censoriously significant to repeat several dataset points as possible, to guarantee that genes and expressed sequence tags labeled as significant are truly significant.

**FIGURE 2:**
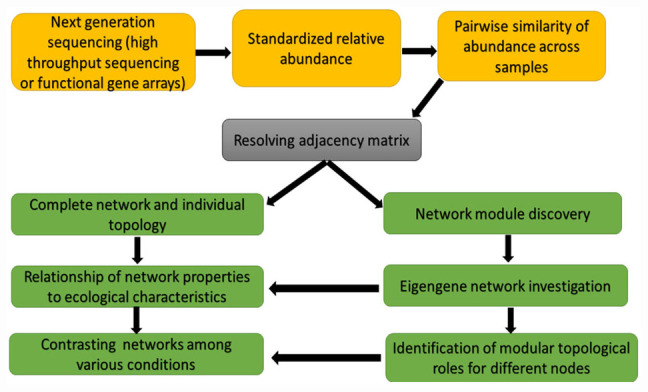
Network construction and network analyses chat based on random matrix theory (RMT)-based approach.

The correlation-based relevance network technique is another method commonly used because the method is straightforward and tolerate noise. The challenge of this method is that their built networks are biased rather than objective due to the used arbitrary thresholds. To resolve this problem, Deng et al. (2012) developed a RMT-based method. This method can robotically find a threshold for cellular network construction from microarray and high-throughput genomics data. The RMT-based approach was useful in a study by Deng et al. (2012) for setting an identical similarity threshold of 0.76, which was short of ambiguity for the phylogenetic molecular ecological networks (pMENs) under warming and unwarming conditions, and guarantees its construction of optimal network. This RMT-based technique (Figure 2) is also useful in identifying and predicting gene function because it is sensitive, fast and robust (Williams et al., 2014). Molecular ecological networks (MENs) resulting from functional gene markers are denoted as functional molecular ecological networks (fMENs; Deng et al., 2012). Although network studies involving plant and plant microbiome researchers dwell more on the pMENs, little or none is known of fMENs in plant-plant microbiome network studies. The fMENs application was employed by Zhou et al. (2010) using a high-throughput functional gene array hybridization dataset of soil microbial communities in a durable grassland-free air and CO_2_ enrichment experiment. Their findings showed that both fMENs under ambient CO_2_ and elevated CO_2_ had the general characteristics (such as modular, small world, scale free, and hierarchical) of complex systems, while the topological structures of the fMENs were dissimilar among ambient CO_2_ and elevated CO_2_ at the levels of the individual functional gene groups, functional genes and the entire communities. This signifies that elevated CO_2_ vividly changed the network connections between diverse microbial functional genes or populations.

Microbial networks regularly comprise many symbiotic components that network in parasitic, commensalistic, mutualistic, ammensalistic, or synergistic modes (Faust et al., 2012). These communications are likely to impact each component’s appropriateness, with straight consequences on soil fertility and plant health (Agler et al., 2016). Around 10 years ago, mutualistic networks have been greatly researched, but the same cannot be said of competitive networks even in microbial ecological studies. This could be due to the following reasons: (i) no network structure is constructed on competitive associations, (ii) in microbial ecology, only limited studies on community scale network have been done, (iii) the absence of experimental data and suitable theoretical frameworks (Deng et al., 2012). Having sound knowledge of these microbe–microbe exchanges is fundamental to envisage the all-inclusive consequences of these communications for plant physiology and wellbeing (Callaway and Howard, 2007). A convenient methodology to increase a better insight of possible communications within the microbial network is to make co-occurrence networks by computing correlations among the richness of individual units (Williams et al., 2014).

Co-occurrence networks aid in recognizing the prospective associations between species, which might be significant for comprehending ecosystem functions and community assembly (Deng et al., 2012). For instance, microbial taxa hypothetically contribute a vital role in the microbiome if they co-occur with other taxa in the co-occurrence networks of the microorganisms. Such microbial taxa are referred to as keystone species, which have a huge controlling effect on their surroundings and other affiliates of the microbiome. In another way round, peripheral species (microbial taxa whose richness does not associate with other microbes) is unaffected in the network by other microorganisms. This means that peripheral species have a lower rate of microbe–microbe interactions compared to keystone species (Barberán et al., 2012). Species assemblies that co-occur share related ecological physiognomies, which can be used to detect traits or microorganisms that are poorly understood (Barberán et al., 2012; Eiler et al., 2012; Sun et al., 2013).

To find topological properties of a network that forecasts keystone species, Berry and Widder (2014) used a generalized Lotka–Volterra dynamics to simulate multi-species microbial communities with recognized interaction patterns. Findings from their research revealed that the number of direct interactions that a keystone species engages in does not increase as keystoneness of a species increases; however, the number of species that are indirectly affected by it increases linearly. Species directly affected by the loss of a keystone had positive interactions with the keystone. Species indirectly affected by keystones, but had an approximately equal number of net negative and positive connections with the keystone species along the most direct path through shared neighbors. Key microorganisms (also known as generalists) in microbial network play vital parts in the network. The more key microorganisms in a network the more ordered and stable a network becomes. This causes a frequent exchange of materials and information among microbial species (Lu et al., 2013).

Network theory, in the form of graph-theoretical methods and systems-oriented, is an amazing approach, which expedite microbial exploration and improve our comprehension of the intricate environmental processes and evolutionary routes involved (Li et al., 2015). Zhang et al. (2018) provided understandings into the organization of soybean rhizosphere microbial communities by implementing a network-based analysis with integrated fungal and bacterial community data to explain the co-occurrence patterns of rhizosphere microbiome in soybean fields. Their observation revealed that in terms of composition and structure, the microbial networks varied among rhizosphere and bulk soil. There were lower modularity, fewer links between fungi and bacteria, and smaller average path length in rhizosphere networks when related to the bulk soil networks. Their work further explained that the northern, southern and global networks of rhizosphere showed lower, higher, and similar complexity, respectively. Soil physicochemical properties such as soil pH and Mg content were reported to greatly influence the variations in the bacterial and fungal sub-networks.

With network theory, microbiome and its entire intricate connections can be modeled and evaluated in a single network (Banerjee et al., 2016). Cooperative metabolic connections point to improved growth of interrelating microorganisms and eventually to positive co-occurrence patterns in abundance, while competition for similar resources point to a counter pattern (Zelezniak et al., 2015; Banerjee et al., 2016). Many patterns reveal the reaction of diverse species to a mutual ecological feature relatively to their direct connections. Thus, a co-occurring microbial pattern could designate they are one or the other; networking synergistically or they have related reactions to ecological features (Barberán et al., 2012; Berry and Widder, 2014; Khan et al., 2019).

When network constructions are made, topological properties need to be measured. Some major properties that can be measured (using tools like Cytoscape) include (i) connectivity of a node to other nodes (i.e., the number of links also called edges), (ii) betweenness centrality (which reflects the number of times a node plays a role as a connector along the shortest path between two other nodes), (iii) clustering coefficient (a degree of interconnectivity in the neighborhood of a node), and (iv) path length (the mean number of edges on the shortest path connecting any two nodes of the network; Khan et al., 2019; Qi et al., 2019). Mean values of these topological properties are employed to define the total structures of the network. The relative betweenness centrality value of each node can designate its comparative significance in the network. Nodes with higher betweenness centrality values are located in the central of the network and those with lower values are anticipated to have a more distant location that signify vital environmental and genetic understandings (Barberán et al., 2012; Berry and Widder, 2014).

In a temperate forest, Toju et al. (2014) evaluate a huge next generation sequencing data of plant–fungus symbiosis by testing if networks of plants and their functionally and phylogenetically diverse root-linked fungi have architectural characteristics that are constant or differ from those of other non-symbiotic and symbiotic networks. Their findings showed that the network of symbiotic interactions among fungal and plant taxa is unequal in species richness (OTUs of plant: fungi = 33:387) and the total network architecture contrasts from that of other ecological networks. However, when they compare the results for other ecological networks and hypothetical expectations for symbiotic networks, the plant–fungus network indicates relatively or adequate low levels of interaction specialization and modularity and a rare form of ‘nested’ network architecture. Several interactions between microbial species assist soil microbes live up to their functions like contributing to nutrient breakdown and redistribution, stimulating plant growth, and subduing pathogens (Faust et al., 2012). Several interactions between microbial species also connote more interchange of metabolites and information between microorganisms, which makes microbial networks perform efficiently.

To use network analysis in identifying robust linkages among microbes inside and between environmental samples, it is important to have relatively comprehensive information on the microbiota present across huge amounts of samples, as without sufficient samples it will be challenging to conclude if co-occurrence patterns are of statistical importance (King et al., 2012; Huang et al., 2020). In a normal sense, the number of samples collected ought to cover four-dimensional or time-based gradients in ecological conditions; this will enhance taxon variability and gives a better meaning to co-occurrence patterns.

The affiliation among diversity and composition of microorganisms in the rhizosphere and plant performance can be negative or positive. An experiment conducted by Maherali and Klironomos (2007) saw that plant well-being improved with increasing mycorrhizal fungal diversity. This shows that diverse functional groups of microorganisms can supplement one other with positive effects on plant growth. This was established from research conducted by Van Der Heijden et al. (2016), where the reported that symbiotic associations between bacteria, fungal, and plant significantly promote plant nutrition, plant biodiversity, and seedling recruitment.

## Functions/Performance of Plant Microbiome

Plant microbiome functions can be beneficial or harmful to plant growth and yield. The functional capacities of plant-associated microorganisms include plant growth promotion, disease symptoms and resistance to biotic and abiotic stress factors (Pérez-Jaramillo et al., 2018). Plant microbiome directly affects some plant functional traits, such as leaf nutrient levels, leaf longevity, specific leaf area, and shoot: root ratio (Berg et al., 2014a). The plant microbiome can determine species coexistence and therefore affect not only a single plant but complete ecosystems (Fitzpatrick et al., 2018).

The mechanism of plant microbiomes in promoting plant growth can be direct or indirect. Direct mechanisms include the production of phytohormones such as auxin, cytokinin, and gibberellin (Compant et al., 2019). These growth hormones modulate endogenous hormone levels in associated plant. Another direct plant growth promoting ability of plant microbiomes is their ability to improve plant nutrient uptake through some biochemical processes such as nitrogen fixation and phosphorus solubilization (Rascovan et al., 2016). Some microbiome such as strains of *Pseudomonas* spp., *Bacillus* spp., *Arthrobacter* can secrete an enzyme called 1-aminocyclopropane-1-carboxylate (ACC) deaminase (Rascovan et al., 2016). This enzyme reduces the level of ethylene (stress hormone) in plants and hence indirectly promoting plant growth by improving plant stress tolerance.

Some plant microbiome such as *Pseudomonas syringae, Erwinia amylovora, Xanthomonas* spp., *Xylella fastidiosa* produce plant toxic compounds proteins, which cause diseases of many crops such as tomatoes, potatoes, green bean, and banana (Mansfield et al., 2012). Plant microbiome has been reported to enhance plant resistance to pathogen infection via commensal-pathogen interactions, the production of antibiotics and pathogen-inhibiting volatile compounds, inducing plant systemic resistance, modulation of plant hormone level, production of lytic enzymes and siderophore (Hopkins et al., 2017; Berg and Koskella, 2018; De Vrieze et al., 2018). Santhanam et al. (2015) and Durán et al. (2018) reported that plant microbiomes improve plant resistance to pathogen infections by mediating disease suppression.

Endophytes have been reported to confer many plant growth-promoting functions on the host plant (Arora and Ramawat, 2017; Fadiji and Babalola, 2020a). They also help in boosting plant growth, yield, and nutrient uptake (Kumar et al., 2017). They have also been reported to perform a key function in pollution control, phytoremediation, and stress tolerance (Su et al., 2015; Karnwal, 2018). A recent report by Fadiji and Babalola (2020a) provided comprehensive details of the antimicrobial/medical functions of endophytes, which include antifungal, anticancer, antimalarial, antituberculosis, antibacterial, antidiabetes, antiarthritic, antiviral, anti-inflammatory effects among others. Some species of *Bacillus*, *Pseudomonas*, and *Arthrobacter* among others have been reported to enhance plant growth via the secretion of ACC deaminase (Kang et al., 2012). Diverse groups of bacteria such as *Paraburkholderia*, *Pantoea*, and *Pseudomonas*, inhabiting the roots of wheat and maize plants have been revealed to possess some plant growth-promoting characteristics such as indole acetic acid production, nitrogen fixation, phosphate solubilization, and ACC deaminase production. Some mechanisms employed in enhancing plant growth include nutrient uptake and stress tolerance (Rascovan et al., 2016; Compant et al., 2019).

A study by Stanton et al. (2014) showed that epiphytes enhanced the water usage of the host plant. Similarly, leaf epiphytic bacteria (*Brickellia veronicifolia)* have been reported to enhance the remediation of air pollutants (Sánchez-López et al., 2018). A recent study also reported the biocontrol activities (antifungal activities) of epiphytic bacterial species of the genera *Acinetobacter*, *Agrobacterium*, and *Burkholderia* from surfaces of red and green pepper (Mamphogoro et al., 2020). Epiphytes are still under investigated, especially in relation to plant growth promotion. Studies establishing the modes of action and roles of these microbial communities in promoting plant growth and health are advocated.

Plants rely on rhizobiome for many biochemical functions, which enhance plant health and growth. Rhizobiome enhances the growth of the plant through the provision of nutrients deficient in the plant and by the secretion of volatile organic compounds, ACC deaminase and plant growth hormones. They also stimulate plant immunity and improve plant health through biocontrol activities by secreting antimicrobial compounds and other mechanisms (Turner et al., 2013; Kwak et al., 2018; Singh et al., 2019). The rhizobiome in most plants is influenced majorly by members of four phyla of bacteria: Proteobacteria, Firmicutes, Bacteroidetes, and Actinobacteria (Niu et al., 2017). Among the four dominated phyla of rhizobiome, Proteobacteria are the most identified groups. Bacteroidetes are involved in denitrification (Van Spanning, 2005). However, Bacteroidetes, Proteobacteria, and Firmicutes serve as copiotrophs, also known as r-strategists, Actinobacteria serves as oligotrophs, also referred to as k-strategists and are also notable producer of many antimicrobial compounds (DeAngelis et al., 2009; Chaparro et al., 2014).

Gene expression across multiple interacting organisms can help understand the complex reality of plant-associated microbiome than observation organisms in isolation. The distinction between the potential roles of plant microbiota and the levels of host interaction as well as the spectrum of these interactions is difficult to understand (Gonzalez et al., 2018). However, metatranscriptomics and metagenomics can help unravel this complexity by allowing gene function to be observed (Gonzalez et al., 2018). Metatranscriptomics gives information about the diversity of active genes within the microbiota, their expression profile and how these levels change due to change environmental conditions.

A study by Saminathan et al. (2018) revealed that the fruit-associated microbiome of different watermelon cultivars were involved in carbohydrate metabolism and ripening of mature fruits. 16S rRNA metagenomics data showed that Proteobacteria and Cyanobacteria were the most abundant phyla in all cultivars, whereas Firmicutes and Bacteroidetes were less abundant in all cultivars tested. The dominance of the Proteobacteria phylum was attributed to their ability to use different carbon sources that help adapt to different environmental changes occurring during fruit development. A reduction in microbial diversity was observed in a cultivar, SDRose. The reduction in diversity was attributed to the expression of peptidoglycan hydrolases associated with pathogenicity of the host plant and high expression of genes linked to infectious diseases. Metatranscriptomic data showed that Proteobacteria was the most abundant bacterial phyla while Ascomycota, Basidiomycota, and Glomeromycota were the fungal phyla identified in all cultivars (Saminathan et al., 2018). Genes involved in amino acid, carbohydrate and energy metabolism, signal transduction and transcription were the most abundant in all cultivars. The study also reported the expression of α-galactosidase genes involved in the process of galactosyl oligosaccharide metabolism (Saminathan et al., 2018).

A comparative metatranscriptomic study on suppression of *Rhizoctonia solani* by wheat rhizosphere microbiome was conducted by Hayden et al. (2018) using two bioinformatics approaches. The study revealed that in *R. solani* suppressive soils, *Stenotrophomonas* and *Buttiauxella* species were the dominant taxa while *Arthrobacter* and *Pseudomonas* species were dominant in non-suppressive samples. The dominance of *Arthrobacter* species in non-suppressive soil was attributed to their ability to degrade cell wall components of wheat, such as cellulose and pectin, and the ability to metabolize wheat root exudates, such as glucose and mannose. In suppressive soils, genes responsible for polyketide and cold-shock stress were more expressed while expressed genes in non-suppressive rhizospheric soils are those responsible for oxidative stress (superoxide dismutase and peroxidases), flagella and antibiotic synthesis (phenazine and pyrrolnitrin). The study attributes the expression of antibiotic genes (phenazine and pyrrolnitrin) in non-suppressive soils by *Pseudomonas* species to defense strategy to mediate competition between *Pseudomonas* and *Arthrobacter* species rather than defense against *R. solani*. These organisms (*Pseudomonas* and *Arthrobacter*) dominate the non-suppressive rhizosphere under conditions of *R. solani* infection because of the ability to survive and function by producing protective reactive oxygen species detoxifying enzymes. However, in the suppressive wheat rhizosphere soil *Stenotrophomonas* species express genes responsible for chemotaxis, polyketide cyclase, superoxide dismutase, fimbrial protein, flagellin, and other biocontrol genes (Hayden et al., 2018).

## Linking Plant Microbiome Composition With Function

The microbes found in the soil are diverse, ranging from bacteria, actinomycetes, viruses, algae, to fungi, nematodes, and protozoa (Geisen et al., 2019). Bacteria occupy a larger proportion of the soil microbes, followed by the actinomycetes, fungi, soil algae, and protozoa in descending order. Each of these organisms or a combined effort of different species determines the overall plant health (Table 1).

**TABLE 1:** Microbiome and their direct positive and negative influence.

Organisms	Species	Activities	Plant Host	References
Bacteria	*Azorhizobium caulinodans*	Nitrogen fixation	*Sesbania*	Ondieki et al. (2017)
	*Sinorhizobium meliloti*	Nitrogen fixation	*Medicago sativa*	Ondieki et al. (2017)
	*Herbaspirillum rubrisubalbicans*	Production of growth regulators	*Saccharum officinarum*	Dos Santos et al. (2017)
	*Soybean Bradyrhizobium japonicum, Bradyrhizobium elkanii*, and *Rhizobium fredii*	Nitrogen fixation	Soy *Glycine max*	Ondieki et al. (2017)
	*Bacillus aryabhattai* and *Pseudomonas auricularis*	Nutrient solubilization	*Camellia oleifera* Abel	Wu et al. (2019)
	*Pseudomonas syringae*	Canker diseases	Kiwi fruit	Wang et al. (2018)
	*Agrobacterium tumefaciens*	Crown gall	*Tectona grandis*	Borges et al. (2019)
	*Erwinia amylovora*	Fire blight diseases	Pears, quince trees, and apple	Doolotkeldieva et al. (2019)
	*Xylella fastidiosa*	Pierce’s diseases	Grape	Overall and Rebek (2017)
	*Ralstonia pseudosolanacearum*	Bacteria wilt	Tomatoes	Klass et al. (2020)
Fungi	*Talaromyces pinophilus*	Production of plant growth-promoting metabolites	Waito-C rice seedlings	Khalmuratova et al. (2015)
	*Microbotryum lychnidisdioicae*	Responsible for anther smut disease	*Silene latifolia*	Kuppireddy et al. (2017); Schirawski and Perlin (2018)
	*Fusarium proliferatum*	Leads to dark brown necrotic spots on leaves and wilting of tomatoes stem	*Solanum lycopersicum*	Gao et al. (2018)
Viruses	Rice necrosis mosaic virus	Plant growth induction and synthesis of metabolite similar to cytokinin	*Ludwigia perennis* and *Corchorus olitorius*	Ghosh et al. (2012)
	Tobacco mosaic virus (TMV)	Mosaic-like mottling discoloration on leaves	Tobacco and pepper	Islam et al. (2018)
	Cucumber mosaic virus	Leaf deformation, curling, and green yellow mosaic	*Pimpinella brachycarpa*	Yoon et al. (2017)
Nematodes	Bacterivorous nematode	Organic matter decomposition and solubility of nutrients	*Lolium perenne*	Gebremikael et al. (2016)
	*Meloidogyne incognita*	Root knot disease	Patchouli	Borah et al. (2018)
	*Aphelenchoides bessseyi*	Green stem and foliar retention	Soybean	Meyer et al. (2017)
Actinomycetes	*Streptomyces rochei* and *Streptomyces thermolilacinus*	Production of plant growth-promoting metabolites and stress tolerance.	*Triticum species*	Jog et al. (2012)
	*Streptomyces coelicolor, Streptomyces olivaceus, and Streptomyces geysiriensis*	Synthesis of siderophore, IAA, and ammonia	*Triticum aestivum*	Yandigeri et al. (2012)
	*Streptomyces ipomoeae a*nd *Nocardia vaccinii*	Sweet potato scab, bud and gall proliferation	Sweet potato andblueberry plant	Anandan et al. (2016)
Algae	*Nostoc*	Indole acetic acid (IAA) synthesis	*Colocasia esculenta* and *Vigna unguiculata*	Ashok et al. (2017)
	*Cephaleuros virescens*	Algal leaf spot	*Manilkara zapota*	Sunpapao et al. (2017)
	*Cephaleuros parasiticus*	Red rust	*Neoregelia Bromeliads*	Sanahuja et al. (2018)

Fungi (mycorrhizal) closely adhere to the roots of plants, in a symbiotic relationship, where the fungi get carbon from the plants and supply needed nutrients to the plant in exchange (Najafi et al., 2012). They have saprophytic abilities and are also capable of causing diseases in plants. Bacteria help decompose wastes and mineralize organic compounds in the soil (Johns, 2017). Actinomycetes resemble both the bacteria and fungi, they have antibiotic properties and as well secrete metabolites that enhance plant growth and drive away pests (Singh et al., 2018). Algae are photosynthetic organisms that live in the soil, enhance the weathering of soil parent material, hold together the soil particles, and when they die, they increase the soil organic matter content (Crouzet et al., 2019). Protozoa are organisms that have antimicrobial properties and help regulate the bacterial population in the soil by feeding on them (Gaines et al., 2019). Viruses and nematodes in the soil have been greatly implicated in plant diseases, making their positive potentials under-utilized. Perhaps, the soil might harbor some viruses and nematodes, which are capable of promoting plant growth. Though some nematodes have been reported to mineralize organic nutrients, which aid plant growth (Gebremikael et al., 2016) and a viral species have been reported to be beneficial to plants (Ghosh et al., 2012; Table 1).

Most soil microorganisms cannot be cultured *in vitro*, therefore making it difficult to properly understand their functions in the soil (Nichols et al., 2008). Bacteria in the rhizosphere region are dominated by *Azotobacter, Serratia, Arthrobacter, Pseudomonas, Rhizobia, Bacillus, Agrobacterium, Mesorhizobium, Enterobacter, Rhodococcus, Burkholderia, Micrococcus, Streptomyces, Alcaligenes, Burkholderia, Cellulomonas, Bradyrhizobium, Azospirillum*, and *Klebsiella* (Prashar et al., 2014). The most abundant rhizospheric fungi include *Fusarium, Trichoderma, Aspergillus*, and *Penicillium* species (Hossain et al., 2017). Other groups of microbes such as archaea (*Candidatus Nitrosoarchaeum koreensis*), viruses (*Rhizoctonia solani* virus), and algae (*Chlorella variabilis* and *Chlamydomonas reinhardtii*) are also present in the rhizosphere (Mendes et al., 2013). The species or family of plants can also determine the type of organism present in the rhizosphere. For instance, Proteobacteria, Bacteroidetes, Actinobacteria, and Acidobacteria dominate the rhizosphere of legumes (Ahrenhoerster et al., 2017; Xiao et al., 2017; Cordero et al., 2020). The rhizosphere of cereals is majorly dominated by Firmicutes, Actinobacteria, Proteobacteria, and Bacteroidetes (DeAngelis et al., 2009; Knief et al., 2012; Bulgarelli et al., 2015; Cordero et al., 2020). Lei et al. (2019) reported the dominance of the members of Sphingomonadales, Xanthomonadales, Rhizobiales, and Burkholderiales belonging to the Proteobacteria phylum as well as the members of the Acidobacteria and Bacteroidetes phylum in the rhizosphere of six plant taxa namely *Ageratum conyzoides, Bidens biternata, Euphorbia hirta, Artemisia argyi, Viola japonica* and *Erigeron annuus.* Direct and indirect activities of microorganisms could have a positive and a negative effect on plants (Olanrewaju et al., 2017; Table 1).

## Positive Interaction

### Direct Microbiome Activities

Direct activities include nitrogen fixation, phosphorus solubilization, and production of cytokinins, ACC deaminase, auxin, and gibberellin (Martínez-Viveros et al., 2010; Olanrewaju et al., 2017). The metabolites released during the direct metabolites have specific functions in plant growth. Auxins help ensure the division of cells, enhance phototropism and geotropism, elongate root and stem of plants, and differentiate vascular tissue (Grobelak et al., 2015). ACC deaminase helps plants resist stress and lower plant ethylene level (Hardoim et al., 2008; Rashid et al., 2012; Glick, 2014). Cytokinins are responsible for regulating cell division, controlling the differentiation of cells in the meristematic tissues of plants, enhancing root elongation, differentiation of chloroplast and xylem, germination of seeds, apical dominance, senescence of leaf, and enhances the proper development of flower and fruits (De Rybel et al., 2016; Olanrewaju et al., 2017; Kieber and Schaller, 2018). Gibberellin enhances flowering, seed germination, setting of fruits, stem elongation (Zaidi et al., 2015), photosynthesis and chlorophyll level (You et al., 2012; Khan et al., 2015).

### Indirect Microbiome Activities

The indirect positive activities of soil organisms refer to the obstruction of pathogenic activities that positively affect plant growth. This can be in the form of competition for space and nutrient, induction of systemic resistance, chelation of Fe, quorum quenching or the production of metabolites (antibiotics, enzymes that degrade the cell wall, hydrogen cyanide, ACC deaminase and siderophore) that hinders their activities or destroy them (Lugtenberg and Kamilova, 2009; Prashar et al., 2014; Olanrewaju et al., 2017).

As the microbes are beneficial to the plants, plants as well have a significant effect on the metabolic activities of microbes. Plants synthesize sugars, amino acids, organic acids, and other metabolites that are used by the microbes as a source of food, them to multiply and perform other metabolic activities (Geetanjali and Jain, 2016). The exudates released by plant root determines the type of microorganisms found in the rhizosphere, the types of microorganism found in turn also modifies the root exudates produced (Prashar et al., 2014). Hence, the belief that microbes are plant specific.

Plants actively recruit beneficial microorganisms to counteract the pathogen assault. This phenomenon known as disease-suppression is a property conferred by resident microbiota. *Serratia* sp., *Bacillus cereus*, and *Bacillus subtilis* have been reported to control a soil-borne disease (phytophthora blight), which affects sweet pepper. This is achieved by increasing the abundance of species such as the *Comamonas, Pontibacter, Sporichthya, Burkholderia, Achromobacter*, and *Ramlibacter* in the rhizosphere, which reduced the population of pathogenic organisms and enhance the chemical parameters (total nitrogen, potassium, ammonia nitrogen, phosphorus, and total organic carbon) of the soil (Guo et al., 2019). Borah et al. (2018) also reported that *Pseudomonas putida* and *Bacillus cereus* enhanced the production of phenylalanine ammonia lyase, while *P. putida* enhanced the production of chalcone synthase, which promoted flavonoids production, consequently having a nematocidal effect. These organisms are actively recruited to counteract the pathogen assault.

### Negative Interactions

The direct mechanism in a negative interaction is the disease-causing ability of microbes. Several microbes are responsible for plant diseases. *Phytophthora capsici* (Rahman et al., 2014), viruses, such as *Cauliflower mosaic virus* (CaMV) and the circulative *Turnip yellows virus* (TuYV; Chesnais et al., 2019), and nematodes, such as cyst nematodes (CN) and root-knot nematodes (RKN; Jones et al., 2013), have been reported to cause plant diseases. The indirect method of plant–microbe negative interaction includes the release of phytotoxins, e.g., ethylene and hydrogen cyanide, which hinders plant root growth (Martínez-Viveros et al., 2010). All these affect the health of plants and reduce plant yield.

A lot has been done on the beneficial activities of bacterial species, especially *Pseudomonas* and *Bacillus*, which has improved their usage. It is therefore necessary to intensify efforts to discover more of the less used soil organism, such as viruses and nematodes (whose harmful effects are more pronounced) for their potential beneficial activities to the plants. Furthermore, the ability to predict the performance of plant due to the microbiome composition in them will go a long way in promoting the role of soil microbiome in agriculture.

## Conclusion and Future Prospects

Currently, interest is growing in studying interactions of plant-associated microbiomes to gain insight into their diverse functions and factors that shaped their functions. These organisms promote plant health and performance under various conditions and can also serve as phytopathogens. With the demand for sustainable crop production, there is growing interest in the exploitation of these microbial functions. Network analysis has shown a formidable potential in establishing the interactions between plant microbiota. Robust networking models are required to study these interactions *in situ*, which is useful in capturing and understanding the interactions between and among plant-associated microbes and changes in the interactions over time. While some of these networking strategies have their limitations, they have answered some key ecological and evolutionary biology questions. We envision that future studies will involve the development of a dynamic network modeling with new experimental designs and current multi-omics techniques that can give a clear perception of the structure, interactions, and functions of these microbiomes as well as the linkages between plant traits and plant microbiota.

## Author Contributions

AA, BE, and OB designed the work. AA, BE, AF, EA, and MA wrote the first draft of the manuscript. AA and OB revised the final draft. OB reviewed the final draft. All authors approved the final draft.

## Conflict of Interest

The authors declare that the research was conducted in the absence of any commercial or financial relationships that could be construed as a potential conflict of interest.
